# Pancreatic and Hepatic Injury in COVID-19: A Worse Prognosis in NAFLD Patients?

**DOI:** 10.3390/biomedicines12020283

**Published:** 2024-01-26

**Authors:** Edgardo Mengual-Moreno, Manuel Nava, Alexander Manzano, Daniela Ariza, Luis D’Marco, Ana Castro, María A. Marquina, Marlon Hernández, Carlos Corredor-Pereira, Ana Checa-Ros, Valmore Bermúdez

**Affiliations:** 1 Biological Research Institute “Doctors Orlando Castejon and Haydee V Castejon”, Universidad del Zulia, Maracaibo 4002, Venezuela; mengual88@hotmail.com; 2 Endocrine and Metabolic Diseases Research Center, School of Medicine, Universidad del Zulia, Maracaibo 4002, Venezuela; manuelnava_14@hotmail.com (M.N.); amanzano_8@hotmail.com (A.M.); arizathings@gmail.com (D.A.); anavaleriacastro@hotmail.com (A.C.); marquinastagg@gmail.com (M.A.M.); marlonjh79@gmail.com (M.H.); 3 Grupo de Investigación en Enfermedades Cardiorenales y Metabólicas, Departamento de Medicina y Cirugía, Facultad de Ciencias de la Salud, Universidad Cardenal Herrera-CEU, CEU Universities, Calle Santiago Ramón y Cajal s/n, 46115 Alfara del Patriarca, Valencia, Spain; luis.dmarcogascon@uchceu.es (L.D.); ana.checaros@uchceu.es (A.C.-R.); 4 Facultad de Ciencias de la Salud, Universidad Simón Bolívar, Barranquilla 080001, Colombia; carlos.corredor@unisimon.edu.co

**Keywords:** COVID-19, SARS-CoV-2, NAFLD, NASH, liver injury, ACE2

## Abstract

The novel disease produced by SARS-CoV-2 mainly harms the respiratory tract, but it has shown the capacity to affect multiple organs. Epidemiologic evidence supports the relationship between Coronavirus Disease 2019 (COVID-19) and pancreatic and hepatic injury development, identified by alterations in these organ function markers. In this regard, it is important to ascertain how the current prevalence of non-alcoholic fatty liver disease (NAFLD) and non-alcoholic steatohepatitis (NASH) might affect COVID-19 evolution and complications. Although it is not clear how SARS-CoV-2 affects both the pancreas and the liver, a multiplicity of potential pathophysiological mechanisms seem to be implicated; among them, a direct viral-induced injury to the organ involving liver and pancreas ACE2 expression. Additionally, immune system dysregulation, coagulopathies, and drugs used to treat the disease could be key for developing complications associated with the patient’s clinical decline. This review aims to provide an overview of the available epidemiologic evidence regarding developing liver and pancreatic alterations in patients with COVID-19, as well as the possible role that NAFLD/NASH might play in the pathophysiological mechanisms underlying some of the complications associated with COVID-19. This review employed a comprehensive search on PubMed using relevant keywords and filters. From the initial 126 articles, those aligning with the research target were selected and evaluated for their methodologies, findings, and conclusions. It sheds light on the potential pathophysiological mechanisms underlying this relationship. As a result, it emphasises the importance of monitoring pancreatic and hepatic function in individuals affected by COVID-19.

## 1. Introduction

SARS-CoV-2, a novel coronavirus, causes the severe acute respiratory syndrome known as Coronavirus Disease 2019 (COVID-19), primarily associated with acute respiratory distress syndrome, which may vary from mild symptoms to lethal pneumonia. It might also present with abdominal pain, nausea, vomiting, and diarrhoea. SARS-CoV-2 has been detected in stool samples of COVID-19 patients, suggesting potential fecal–oral transmission [1].

Furthermore, elevated alanine transaminase (ALT), aspartate transaminase (AST), and total bilirubin (TB) levels have been found in 15–16% of COVID-19 patients [2], with 44% showing abnormal liver function tests. These parameters were significantly increased in severely ill patients [3,4]. Likewise, clinical manifestations, imaging findings, and pancreatic enzyme alterations support pancreatitis diagnosis in 0.27% of patients with COVID-19 [5].

These findings might result from increased caloric intake and sedentarism in modern Western society [6,7], associated with hepatic fat accumulation in certain diseases, such as non-alcoholic fatty liver disease (NAFLD). NAFLD is characterised by fat accumulation in more than 5% of hepatocytes without other causes. NAFLD is often associated with insulin resistance and metabolic syndrome. Histopathological manifestations can range from benign hepatic steatosis to non-alcoholic steatohepatitis (NASH), which may finally lead to cirrhosis [8,9].

Due to SARS-CoV-2 infection to intestinal cells and the altered hepatic and pancreatic function tests, several studies have explored the possible effect of the virus on the presentation, evolution, and clinical outcome [3,10,11,12,13,14]. However, there is scarce information on the real effect of SARS-CoV-2 on the liver or the pancreas. This review aims to provide the potential pathophysiology and epidemiological association between COVID-19 and pancreatic and liver injury, particularly in patients with pre-existing NAFLD/NASH.

In this review paper, the methodology involved a comprehensive search of the PubMed database using relevant keywords and Boolean operators. The search strategy included filters to retrieve the most relevant articles, such as publication date and study types. One hundred thirty-two articles were selected based on their alignment with the research question and contribution to the topic. The full texts of these articles were carefully evaluated to understand their methodologies, findings, and conclusions.

## 2. Pancreatic Injury in COVID-19

### 2.1. Epidemiological Evidence of Pancreatic Injury

Several case reports have documented acute pancreatitis (AP) in COVID-19 patients who presented pancreatic-type abdominal pain, high serum amylase/lipase levels, or characteristic imaging findings (Table 1). One study reported the case of a 49-year-old woman with COVID-19-associated symptoms who complained of severe epigastralgia radiating to her back on the second day after hospital admission [15]. She also showed increased pancreatic enzymes and changes in abdominal computed tomography (CT). The diagnosis of AP due to COVID-19 was confirmed after thoroughly ruling out other potential causes [15]. Alves et al. [16] described a 56-year-old woman with COVID-19 whose CT scan exhibited pancreatic abnormalities: amylase and lipase levels that increased from 249 to 544 U/L and 580 to 2993 U/L, respectively. These alterations appeared seven days after hospitalisation and without relevant abdominal symptoms. Despite a modified Glasgow score of 3, no complications were identified, and the only treatment was fluid therapy and a bland diet [16].

Pancreatic necrosis was also reported in a 67-year-old woman with epigastralgia, diarrhoea, and vomiting. Upon admission, the diagnosis of COVID-19 was confirmed, and abdominal and pelvic CT scans were requested. The CT showed no enhancement of the pancreatic head and body, indicating necrotising pancreatitis; an abdominal ultrasound did not detect gallstones. Her serum amylase levels rose to 1483 U/L [17]. After discarding several possible pathologies, acute necrotising pancreatitis was diagnosed. Comparable findings were reported by Rabice et al. [18] and Cerda-Contreras et al. [19], who documented clinical and paraclinical characteristics of AP in a pregnant patient and an elderly woman with COVID-19, respectively. However, no direct evidence of viruses commonly associated with pancreatitis or biliary stones exists, so COVID-19-induced pancreatitis should be considered.

On the other hand, a prospective study on 316 patients determined that AP was not present in mild cases of COVID-19 pneumonia. However, it was present in 32.5% of critically ill patients. Furthermore, hospitalisation and mortality rates were higher in patients with COVID-19 accompanied by AP (*p* = 0.0038 and *p* < 0.0001, respectively) [20].

A systematic review, which included 11 patients with COVID-19 and AP, showed that only five patients had AP as per the Atlanta classification. Most cases were considered to be induced by SARS-CoV-2; nevertheless, other established etiological factors were overlooked [21].

More epidemiological evidence was reported in other observational studies, where patients presented high serum amylase/lipase levels and unexplained glucose intolerance (Table 2). Wang et al. [13] reported that of 52 patients with COVID-19 pneumonia, 17% of patients had pancreatic injury, defined as any abnormality in amylase or lipase levels. Similarly, Bruno et al. [14] observed that 6 of the 70 patients with COVID-19 included in their study had abnormal pancreatic parameters. In one of the patients, these abnormalities were present at the time of admission, while the other five developed enzymatic alterations during hospitalisation. A multicentric study, which included all COVID-19 patients with quantified serum lipase levels, showed that out of the 71 patients included, 9 (12.1%) developed hyperlipasemia > 60 U/L upon admission. Only two patients (2.8%) developed hyperlipasemia > 3 × upper limit of normal (ULN), i.e., >180 U/L [22].

Another study that included 83 hospitalised COVID-19 patients found that 14 patients (16.8%) developed hyperlipasemia > 3 × ULN during their stay at the ICU (32.8% at admission vs. 92.9% two weeks later; *p* < 0.001) and higher intubation frequency (78.6% vs. 23.5%; *p* = 0.002) than those without hyperlipasemia [23]. In this subject, Rasch et al. [24] reported, in a different study, that from 38 COVID-19 patients admitted to ICU, 10 patients (26%) had serum lipase levels around 60 and 180 U/L (mean 104.5 ± 29.6), and 12 patients (31.6%) developed hyperlipasemia > 3 × ULN. Despite this, no association was found between lipasemia > 180 U/L and specific outcome parameters, such as ventilation duration or mortality. It is crucial to indicate that in 5 out of these 12 patients (41.7%) with lipasemia > 3 × ULN, the presented increase occurred after the viral clearance. Moreover, in a retrospective study performed on nineteen patients admitted to ICU due to COVID-19, Zhang et al. [25] pointed out that serum amylase levels increased in all patients, punctually 65.7 mg/L (56.9–100.8 mg/L) among survivors, and 95.1 mg/L (54.8–79.8 mg/L) among non-surviving patients. 

However, to conclusively diagnose AP, two of the three traditional criteria by the Revised Atlanta Classification (pancreatic type abdominal pain, serum amylase/lipase levels three times higher than the ULN, or characteristic imaging findings) must be met [26]. Even though serum amylase/lipase levels were undoubtedly elevated in the reported studies, they did not rise over or even to the expected three times ULN figures in most cases, and only some authors reported conclusive imaging findings. While similar in their results, the studies have differences in their methodologies, leading to interpretation conflicts, especially since they define hyperlipasemia based on different reference values. 

### 2.2. SARS-CoV-2 Infection and Endocrine Pancreas: A New Cause of Diabetes Mellitus? 

Several reports show that SARS-CoV-2-infected patients can exhibit blood glucose alterations. This finding is expected due to the virus’s potential impact on the endocrine pancreas [27]. Although serious diseases might be associated with stress-induced hyperglycemia, Yang et al. [28] described SARS (caused by SARS-CoV) patients who showed fasting plasma glucose levels significantly higher than those without SARS but likewise underwent pneumonia. Patients with a history of diabetes who later developed COVID-19 presented uncontrolled hyperglycemia and episodes of acute hyperglycemic crises that required exceptionally high insulin doses [29,30]. Both diabetic ketoacidosis and hyperglycemic hyperosmolar state are common among COVID-19 patients with diabetes, becoming a clinical emergency [31,32].

Glucocorticoids are potent anti-inflammatory and immunosuppressive agents commonly used in hospitalised COVID-19 patients [33]. Despite decreasing COVID-19 mortality, steroids tend to elevate glycemia levels [34]. It has been reported that approximately 40% of inpatients consulting the endocrinology service are due to new onset steroid-induced diabetes, or type 2 diabetes exacerbated by steroid use [35]. Similar to other hospitals, in which 53–70% of individuals with no history of diabetes develop steroid-induced hyperglycemia [36], Keerthi et al. [37] report that 75% of patients who developed new-onset diabetes mellitus were treated with steroids as part of their treatment for COVID-19. In consequence, steroid use in COVID-19 patients has been linked to worse glycemic control, requiring strict glycemic control. Therefore, for patients with COVID-19, rigorous glycemic control becomes essential. [33].

Finally, recent evidence shows that newly diagnosed diabetes is common in COVID-19 patients [30]. For this reason, Sathish et al. [38] performed a systematic review and a meta-analysis that included eight studies totalling 3711 COVID-19 patients, among whom there were 492 cases of newly diagnosed diabetes. The random-effects meta-analysis estimated a combined proportion of 14.4% (95% CI: 5.9–25.8%) with a high degree of heterogeneity (I^2^: 98.6%, *p* < 0.001), leaving open the probability that SARS-CoV-2 infection could develop diabetes. These studies suggested that more cases worldwide are needed to ascertain if this novel virus triggers diabetes development.

### 2.3. Pathophysiological Mechanisms

An early finding was that SARS-CoV-2 virus attached to the cell surface angiotensin-converting-enzyme 2 (ACE2) to enter the cells. The virus spike protein (S) recognises a specific region of ACE2 known as the ACE2 receptor. The receptor is mainly found in lung tissue and, to a lesser extent, in intestinal, hepatic, cardiovascular, renal, adipose, and pancreatic tissues, supporting the theory that the virus can have multi-organic effects [39]. The protein S must be cleaved to sites S1/S2 and S2’ to enter the cytoplasm. A cellular serine protease, TMPRSS2, performs this cleavage, allowing viral and cellular membrane fusion and entry into the cell. For this reason, both ACE2 receptor and TMPRSS2 expression are needed for SARS-CoV-2 to access target cells [40], expressed in the alveolar tissue and the pancreatic β cells [12].

Additionally, ACE2 plays a crucial role in body homeostasis and how ACE2/angiotensin 1–7 stimulates insulin secretion and reduces insulin resistance. It is important to note that there have been reports on downregulated ACE2 expression in SARS-CoV infection. ACE/ACE2 imbalance results in blood pressure dysregulation, systemic inflammation, and other unknown effects on pancreatic function [41].

The genetic similarity between SARS-CoV and SARS-CoV-2 suggests that ACE2 expression downregulation in SARS-CoV-2 infection could be linked to the multiple organ dysfunction syndrome described in COVID-19 [42]. Furthermore, the accumulation of angiotensin II in COVID-19 may affect pancreatic homeostasis, contributing to insulin resistance, hypoxia, and extracellular acidification via pancreatic Na+/H+ exchanger activation. Moreover, this damage is exacerbated by the increase in lactate concentration released from the adipose tissue in COVID-19 [43].

The current understanding of SARS-CoV-2-related pancreatic injury comes from earlier SARS-CoV research and theoretical reasoning supported by epidemiological evidence [28,44]. Therefore, no definitive causal pathophysiologic mechanism has been identified, and the heterogeneous diagnostic criteria for acute pancreatitis have resulted in irregular results [21].

Previous studies on SARS-CoV suggest mechanisms for SARS-CoV-2-mediated AP. SARS-CoV has been found in the pancreatic tissue of patients who died from SARS [12,45]. In this regard, there is a slightly higher amount of ACE2 receptors in the pancreatic tissue than in the lungs of healthy subjects [10,36]. ACE2 receptors in pancreatic exocrine and endocrine cells permit not only SARS-CoV-2 entry but replication within the cells [46]. However, it remains unclear whether pancreatic injury is solely due to local viral replication or if this is a condition for further damage leading to AP.

**Figure 1:**
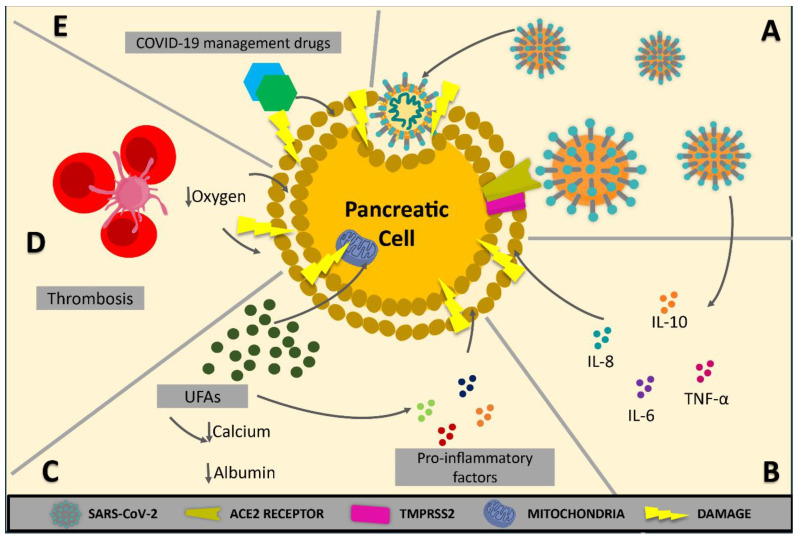
Pathophysiological mechanisms of pancreatic injury in COVID-19. Although more research is still needed to completely understand the underlying mechanisms linking COVID-19 and pancreatic injury, various theories have been proposed thus far. (**A**) Direct and indirect injury due to viral entry and replication. Pancreatic cells possess many ACE2 receptors that, coupled with the TMPRSS2 protease, make viral entrance possible. Subsequent viral replication within the cells could lead to cytopathic effects while indirectly interacting with other factors that could result in pancreatic injury. (**B**) Cytokine-mediated damage. Immune dysregulation is key in severe cases of COVID-19, similar to severe cases of acute pancreatitis. Both pathologies have shown an unrestrained immune response that leads to direct and indirect immune-driven cellular damage. (**C**) Unsaturated fatty acid (UFAs)-mediated damage. A sudden spike in UFA levels can result in mitochondrial damage, hypocalcemia, and hypoalbuminemia while also, directly and indirectly, mediating pro-inflammatory effects within the system that can lead to the trigger of a cytokine storm. Elevated UFA levels have been observed in both COVID-19 and acute pancreatitis, which points to a common pathophysiological mechanism. (**D**) Thrombotic and microvascular-mediated damage. It has been reported that SARS-CoV2 can cause severe endothelitis, coagulopathies, and thrombosis, which can end in micro-ischemic events affecting pancreatic tissue and causing severe injury. Moreover, septic shock causes gastrointestinal hypoperfusion and acute ischemic pancreatitis; this, coupled with SARS-CoV2-mediated hypoxia, gives further weight to this theory. (**E**) Drug-mediated damage. Some drugs commonly used to manage COVID-19, such as NSAIDs, steroids, and tocilizumab, have been reported to directly or indirectly cause acute pancreatitis.

Severe COVID-19 often presents with immune dysregulation, cytokine storm, and systemic inflammation, which could indirectly result in pancreatic injury [47,48]. A recent cytokine profile found in both severe AP and COVID-19 cases showed significantly higher levels of IL-6, IL-8, IL-10, and TNF-α in the severe cohort of both diseases in comparison with their non-severe counterparts, suggesting they might share an underlying common pathophysiological mechanism (Figure 1) [49]. Mainly, IL-6 levels have been linked to disease-related mortality in COVID-19-induced acute respiratory distress syndrome (ARDS) [50] and the risk of severe pancreatitis [51].

Other relationships between AP and COVID-19 have been described. Severe cases of AP might result in multisystem organ failure (MSOF), ARDS, and death [52]. These features are related to a sudden increase in unsaturated fatty acids (UFAs) driven by pancreatic triglyceride lipase-mediated adipocyte lipolysis of visceral adipose tissue [51]. UFAs, which have a pro-inflammatory effect, can lead to death due to their ability to cause mitochondrial damage and cell apoptosis [53].

The involvement of early and progressive hypocalcemia and hypoalbuminemia in severe COVID-19 could be due to UFAs binding to both albumin (ALB) and calcium. Supplementation in the early stages of COVID-19 could reduce lipotoxic MSOF, but not once MSOF has set in [54].

SARS-CoV2 causes severe endothelins, coagulopathies, and thrombosis, which could cause microischemic events and severe pancreatic injury [55,56,57]. Gastrointestinal hypoperfusion in septic shock and some cases of ischemic AP have been reported [58,59]. Similarly, AP is directly related to the activation of coagulation cascades. Excessive inflammation, added to platelet activation and endothelial dysfunction in COVID-19, might lead to venous thromboembolism and other related hemostasis issues, contributing to pancreatic injury [42]. However, further research in this area is needed.

Finally, drugs, including NSAIDs, steroids, and tocilizumab, are a significant factor in the multifactorial pathophysiology of pancreatic injury, adding complexity to the diagnosis of SARS-CoV-2 involvement in AP aetiology [60,61,62]. Several reports have been published showing that these drugs may directly or indirectly result in AP [63,64,65,66].

SARS-CoV-2 could theoretically cause pancreatic injury, but there is little significant epidemiological evidence to support this theory. Thus, further investigation is required to obtain impactful data that could establish a solid causal relationship.

## 3. Hepatic Injury in COVID-19

### 3.1. Epidemiological Evidence of Hepatic Injury

Several systematic reviews and meta-analyses about the association between COVID-19 and abnormal liver function tests (LFTs) have been published. These studies agree that some liver tests may be altered in severe COVID-19 cases, including those that estimate biosynthetic function (ALB; prothrombin time), hepatic clearance/biliary secretion capacity (BIL: bilirubin), and other markers of liver or biliary injury (GGT: gamma-glutamyltransferase; ALP: alkaline phosphatase). However, it is essential to note that certain studies did not specify whether patients had pre-existing liver injury or chronic liver disease, which may have influenced the reported results. These results are summarised in Table 3.

Ahmed et al. [67] published the results of a meta-analysis that included 8817 patients. The accepted parameters for liver injury were defined as levels of transaminases > 3 × upper limit of normal and higher than upper normal levels of ALT, AST, and BIL. They found a prevalence of 15.7% (9.5–23.0%) in ALT, AST, and BIL, significantly higher in patients with severe COVID-19. Serum ALB levels were considerably lower than in non-severe cases. They also found that liver injury extent was associated with COVID-19 severity. Abdulla et al. [68] and Parohan et al. [69] equally reported that liver biomarker levels seemed higher in patients with severe COVID-19, which could serve as prognostic factors for estimating disease severity. Furthermore, Wu and Yang [70] determined that the alteration of liver parameters increased by 1.98 times the mortality risk in COVID-19 patients. Shokri Afra H. et al. [71] found this association more frequent in male patients, whereas Youssef et al. [72] showed that COVID-19 severity was linked to prolonged prothrombin time.

Other findings indicate that the prevalence of abnormal LFTs could vary according to the disease progression. Within this context, Wu et al. [73] investigated LFT alterations in COVID-19 patients upon admission and during hospitalisation. Abnormal LFTs were found in 27.2% of patients upon admission and 36% during hospitalisation. Lower levels of albumin (39.8%), GGT (35.8%), AST (21.8%), and ALT (20.4%) were observed upon admission. During hospitalisation, the most common LFT abnormalities were ALT (38.4%), AST (28.1%), and BIL (23.2%). Kumar-M et al. [10] found different percentages: hypoalbuminemia (61.27%), increased GGT (27.94%), ALT (23.28%), and AST (23.41%). Moreover, they also reported that the prevalence of acute liver injury was 23.7%, and the risk of developing it was up to two times higher in patients with severe COVID-19 (RR = 2.18; 95% CI: 1.49 to 3.18; *p* < 0.05; I^2^ = 67%).

However, it is crucial to consider that many of these parameters are not specific to the liver. This means their increase could also be secondary to damage in other organs or tissues, e.g., skeletal muscle [74].

### 3.2. Pathophysiological Mechanisms

**Figure 2:**
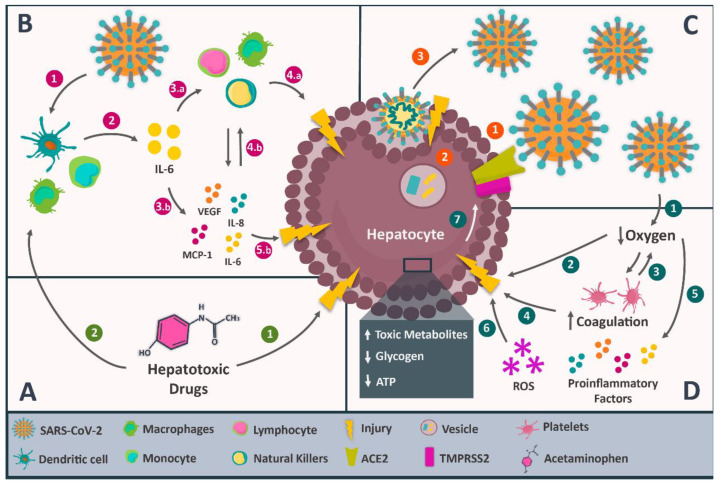
Pathophysiological mechanisms of liver injury in COVID-19. Within the diverse proposed mechanisms, four stand out, starting with the hepatotoxic effects of drugs used for COVID-19 treatment, which are indicated in rectangle (**A**); this mechanism is divided into (1) the direct toxic effect of the drugs on liver cells and (2) the indirect effect of the drugs by inducing an immunological response. On the other hand, rectangle (**B**) indicates the dysregulated immune response, which begins by (1) the SARS-CoV-2 infection on dendritic cells, monocytes, and macrophages, which then (2) triggers the production of interleukin 6 (IL-6), a cytokine that acts in two ways: first (3.a), it activates immune cells, such as lymphocytes, macrophages, and natural killers capable of (4.a) injuring hepatocytes; and secondly (3.b), it stimulates the secretion of the vascular endothelial growth factor (VEGF), monocyte chemoattractant protein-1 (MCP-1), IL-8, and additional IL-6, (4.b) increasing the activity of immune cells and (5.b) causing damage in the hepatocytes. Likewise, the direct viral damage indicated in rectangle (**C**) starts with (1) the interaction of SARS-CoV-2 with the hepatic ACE2 and TMPRSS2, enabling (2) the viral entry and replication inside the hepatocytes, which (3) produce a cytopathic effect and damages the cell. Finally, rectangle (**D**) indicates the hypoxic and ischemic liver injury, which begins with (1) the SARS-CoV-2 induced hypoxemia, (2) affecting the liver cell function by inducing anaerobic metabolism, along with the accumulation of toxic metabolites and depletion of the cell energy, entailing cell injury; in addition, (3) hypoxemia also affects coagulation and platelet activity, favouring thrombus formation and ischemic events that (4) injure liver cells as well; this is accelerated by reactive oxygen (5) species and the release of pro-inflammatory factors, which will contribute to the (6) hepatocyte injury and, therefore, (7) an increase in the expression of ACE2.

Identifying the cause behind liver injury in COVID-19 has become a promising area of research, considering its impact on the clinical evolution of patients. Several mechanisms have been proposed. Among them are direct viral damage, dysregulation of the immune response, hypoxic and ischemic liver injury, and drug-induced liver injury during COVID-19 treatment, without excluding a possible multifactorial aetiology (Figure 2). To elucidate the possible contribution of this unfamiliar disease to liver injury, it is necessary to review all possible mechanisms proposed in the literature [75,76].

#### 3.2.1. Direct Viral Damage

Recent studies have posed direct viral damage as a pathophysiological mechanism of hepatic injury in COVID-19. This proposal is based on SARS-CoV2 in the livers of infected patients and the alterations in hepatic function. Tian et al. [77] reported SARS-CoV-2 RNA sequences in liver tissue via RT-PCR. Wang et al. [78] identified coronavirus particles inside the cytoplasm of hepatocytes in patients with elevated aminotransferases. Other studies reported that ultrastructural characteristics and biopsies indicate a typical direct viral-mediated injury.

For this pathophysiological mechanism to work, the presence of the ACE2 receptor and TMPRSS2 on the hepatocyte surface is required [78,79,80]. SARS-CoV-2 will bind to the ACE2 receptor through the S protein, and this would trigger TMPRSS2 expression on the hepatocyte membrane, the internalisation of the viral particle and its further replication, cell busting, viral release, and hepatic injury, as reported by Wang et al. [78]. However, reports on ACE2 receptor expression in the liver are limited. The Human Protein Atlas indicates its virtual absence in the liver, but Li et al. [80] found medium ACE2 expression levels in liver tissue, similar to the lung. Chai et al. [81] also confirmed ACE2 presence in the liver, with lower expression in hepatocytes than in cholangiocytes. They proposed that SARS-CoV-2 could infect and injure bile ducts, explaining liver alterations. This aligns with increased bilirubin and GGT levels in COVID-19 patients. Notably, basal gene expression does not measure ACE2 molecules on the cell membrane [81,82,83].

#### 3.2.2. Dysregulation of the Immune Response

A meta-analysis reported by Zeng et al. [84] pinpointed the association between the presence of inflammatory markers and COVID-19 severity. Li et al. [85] showed that lymphopenia and C-reactive protein levels ≥ 20 mg/L are independent risk factors of liver injury in patients diagnosed with COVID-19, similar to previous findings in SARS-CoV and MERS-CoV-infected patients. In those patients, levels of inflammatory markers were higher in cases with altered liver function compared with those with normal liver function [84,85,86,87].

Liver biopsies of COVID-19 patients revealed hepatomegaly, hepatocyte degeneration, lobular necrosis, neutrophil infiltration, lymphocyte and monocyte infiltration in the portal area, sinusoidal congestion, and microthrombosis. These findings suggest immunological hyperactivity and may explain reported cases of lymphopenia. The exact mechanism of immune system-induced liver injury is unclear. However, based on the medical history of SARS-CoV and MERS-CoV patients, the cytokine storm has been proposed as a major contributing factor, similar to cytokine storm syndrome observed in COVID-19 patients [88,89,90].

Thus, the cytokine storm could be responsible for the liver alterations through a hypothetical mechanism triggered by the SARS-CoV-2 invasion of monocytes, macrophages, and dendritic cells. The invasion would lead to the synthesis of pro-inflammatory interleukins, especially IL-6, which might affect innate and adaptive immune system cells, leading to hyperactivation. Eventually, all this exaggerated immunological response could injure the liver tissue and lead to observable clinical symptoms [91,92,93].

#### 3.2.3. Hypoxic and Ischemic Liver Injury

It is important to note that the most frequent symptom of COVID-19 is dyspnea, which can be accompanied by different hypoxemia degrees. This alteration is usually associated with coagulation and platelet activity alterations, producing thrombosis and triggering ischemic events [94,95,96]. There are occasional reports of liver necrosis, which could be attributed to ischemia [10,77].

Concerning the liver, hepatocytes are affected by lipid accumulation and glycogen and ATP depletion, characteristic of anaerobic metabolism. There is also toxic metabolite accumulation and inhibition of cell survival pathways, resulting in hepatocyte death. The production of reactive oxygen species and the liberation of pro-inflammatory factors exacerbate the situation. Hepatocyte death leads to liver function alterations, which tend to increase ACE2 expression, consequently magnifying the direct viral damage to the liver [82,87,97].

#### 3.2.4. Drug-Induced Liver Injury during the Treatment of COVID-19

Several drugs used to treat COVID-19 are known to have potential hepatotoxic effects and could be responsible for, or at least contribute to, liver alterations. Acetaminophen is a widely used analgesic and antipyretic drug for COVID-19 [11,76,98,99]. Eassawy et al. [99] confirmed this finding by treating mice with high doses of acetaminophen, demonstrating a corresponding increase in liver aminotransferases, usually higher in cases of liver injury in COVID-19. However, great caution must be exercised in translating animal findings to human beings [11,98].

Another drug involved is tocilizumab. Muhović et al. [100] reported, for the first time, a case of liver injury linked to the use of tocilizumab in a patient with COVID-19. They described that, two days after receiving two doses of 400 mg of tocilizumab, the patient started to show altered levels of both aspartate transaminase and alanine transaminase. Falcão et al. [101] associated the use of hydroxychloroquine with increased serum transaminase levels in a 29-year-old woman with COVID-19. The transaminase levels returned to normal five days after discontinuing the drug.

Cheng et al. [102] studied the effect of lopinavir, ritonavir, and abidol, three antiviral drugs commonly included in the treatment of COVID-19, on 134 patients with SARS-CoV-2 pneumonia, divided into three groups: the first group, with 52 patients, received lopinavir/ritonavir; the second group (34 patients) received abidol; and the third, with 48 patients, was the control group. They found that two patients in each group developed a mild liver injury. In another study, Zampino et al. [103] described five cases of hepatotoxicity in patients with COVID-19 treated with remdesivir. They showed decreased bilirubin levels and increased serum transaminase levels. No prior history of liver injury was present in the patients, and all liver function tests were normal before drug administration.

The hepatotoxic effect of drugs can be due to the drug itself or the immune response. Acetaminophen, for instance, induces liver injury when metabolised in the liver through the cytochrome P450-detoxification chain. When the liver’s detoxification capacity is exceeded, it causes oxidative stress, mitochondrial dysfunction, and cell death, which can occur via apoptosis or necrosis. The immune response also plays a role, as drugs can bind to Toll-like receptors, activating macrophages, natural killer cells, and cytokines, potentially leading to cell death. Drugs or their metabolites acting as haptens can also trigger a signalling cascade in T-CD4+ lymphocytes, resulting in liver cell apoptosis. [104,105].

The most probable mechanism is multifactorial. There is evidence of an increase in the expression of ACE2 in injured liver tissue, caused by a dysregulated immune response against SARS-CoV-2, ischemia or hypoxia during the clinical evolution of COVID-19, or by hepatotoxic drugs used during the treatment [10,81,82,97].

## 4. Hepatic Injury in COVID-19 Patients with Pre-Existing NAFLD/NASH

Retrospective studies have determined that 30.7% of patients hospitalised for COVID-19 presented with NAFLD as per the hepatic steatosis index (HIS). They also were at higher risk of developing liver damage when infected with the virus in comparison with patients without NAFLD [6]. In another study conducted in Qatar, 589 patients were divided into two groups: NAFLD and non-NAFLD. The authors found that NAFLD was an independent predictor of mild or moderate liver injury in hospitalised COVID-19 patients [106]. Similarly, Hashemi et al. [107] compared COVID-19 adults with and without NAFLD and found that the former were more likely to be admitted to the ICU and require mechanical ventilation. However, only those with cirrhosis had an increased mortality risk.

Ji et al. [108] studied 202 patients with COVID-19 and previous NAFLD. Liver injury was observed in 50% of these subjects upon admission and 75.2% throughout hospitalisation. Statistical analyses revealed that patients with NAFLD had a higher risk of disease progression, a higher probability of abnormal liver function, and a longer virus clearance time than healthy patients. A recent study by Yao et al. [109] on individuals with NAFLD and COVID-19 found that concurrence of diabetes and advanced liver fibrosis was an independent risk factor for severe disease.

NAFLD or NASH are entities frequently clustered with obesity, metabolic syndrome, dyslipidemia, diabetes, and hypertension, all linked to COVID-19 severity [110]. NAFLD/NASH are also related to metabolically active fat expansion, insulin resistance, hepatic accumulation of lipids, and a chronic pro-inflammatory state, which can be concomitant and interact with SARS-CoV2-induced acute inflammation, worsening NAFLD/NASH and COVID-19 [111].

Hepatocytes can release stress/danger signals in response to hepatic lipids accumulation, triggering sterile inflammatory pathways through caspase and TUNEL-positive cell activation, driving chronic injury and cell death in the long term. At the same time, hepatocyte stress and death lead to damage-associated molecular patterns (DAMPs) release, which in turn activate liver immune cells, like Kupffer cells and stellate cells; the former produce pro-inflammatory cytokines, thereby contributing to hepatocyte injury and death, and consequently, hepatic fibrosis [112].

Many pro-inflammatory markers increase during COVID-19, including IL-6 and MCP-1, which are key factors for the cytokine storm and acute inflammation caused by COVID-19 and contribute to chronic inflammation caused by NAFLD/NASH. The release of IL-6 causes immune cell hyperactivation and the secretion of cytokines like MCP-1, increasing the recruitment of Kupffer cells and, thereby, exacerbating steatohepatitis [112,113].

Similarly, within the pathogenesis of NAFLD, the innate immunity response is characterised by the polarisation of macrophages towards the M1 phenotype as a consequence of the pro-inflammatory activity. This phenomenon is characterised by the decreased activity of anti-inflammatory M2 macrophages, which could contribute to COVID-19 progression. This phenomenon would be achieved through the stimulation of macrophages by metabolic agents, such as lipopolysaccharides (LPS), signals from the intestine-liver axis (dysbiosis and endotoxins), and low-grade systemic inflammation (TNFα), being stimuli characteristic of obesity, diabetes, and metabolic syndrome [114].

Once M1 macrophages have been activated, they will rely on aerobic glycolysis to satisfy the increased need for ATP and metabolic precursors, with hypoxia-inducible factor (HIF)-1α being a key element. LPS and TNFα activate HIF-1α by elevating the levels of succinate, an intermediate of the citric acid cycle. Succinate, in turn, inhibits oxygen-sensitive prolyl hydroxylases, leading to pseudohypoxia and stabilisation of HIF-1α; subsequently, the latter increases the expression of the glucose transporters GLUT-1 and GLUT-3. However, once glucose enters the cell, it will be converted into lactate instead of entering the Krebs cycle as pyruvate. HIF-1α also upregulates the expression of the enzyme pyruvate-dehydrogenase kinase and shunts glucose metabolism towards glycolysis, away from oxidative phosphorylation [115].

At the same time, HIF-1α directly promotes M1 gene expression. By increasing the number and activity of M1 macrophages, HIF-1α also elevates their production of transforming growth factor β, IL-1β and TNF-α. These mediators promote hepatic stellate cell survival, as well as their activation and differentiation to myofibroblasts, which are vital in NAFLD/NASH liver fibrosis [115,116,117]. This point highlights that underlying liver fibrosis could represent an independent risk factor for severity in patients with COVID-19 [118].

Obesity is a risk factor for developing NAFLD/NASH; severe COVID-19 infection is another significant component in this inflammatory process. In fact, in obesity, the storage capacity of the adipose tissue is exceeded, triggering the release of stress signals and activating the previously described inflammatory pathway [112,119]. On the other hand, obesity plays a role in this process by producing pro-inflammatory cytokines that promote steatosis, fibrosis, and hepatic inflammation [120].

Likewise, it has been observed that SARS-CoV-2 is capable of causing microvesicular and macrovesicular steatosis through lipid metabolism and mitochondrial activity dysregulation. The latter is responsible for causing microvesicular steatosis due to defects in the mitochondrial β oxidation. COVID-19 and NAFLD/NASH share this defect, potentially leading to a compounded impact in patients diagnosed with both. This joint impact may arise due to the SARS-CoV-2 direct effect, cytokine storms, and drug side effects, such as corticosteroids [121,122].

At the same time, SARS-CoV2 can indirectly injure the liver through an uncontrolled immune response, hypoxia, or the hepatotoxic effect of administered drugs, as previously described. Furthermore, patients with NAFLD/NASH are more susceptible to drug-induced liver damage, leading to NAFLD/NASH worsening [123,124].

## 5. Discussion

In pancreatic injury, substantial evidence indicates that the pathophysiological processes associated with COVID-19 could impact the pancreas. Alterations in pancreatic enzyme levels were found in certain COVID-19 patients, which correlated with structural changes in the pancreas, as shown by imaging studies [13,14,18,19,22,23,24,25]. The appearance of diabetes throughout COVID-19 infection was also reported in several patients [30,38]. However, whether this endocrine pancreatic alteration was a direct consequence of SARS-CoV-2 infection remains inconclusive. This damage to the pancreas could result from local viral replication of SARS-CoV-2, leading to direct cellular damage [46], or from the systemic pro-inflammatory state characteristic of the disease. Notably, elevated levels of IL-6 were associated with a higher risk of mortality and the development of acute pancreatitis in COVID-19 patients [125]. UFAs-mediated lipotoxicity [53], endothelial dysfunction, and alterations in the coagulation cascade due to excessive systemic inflammation [42] appear to contribute to developing pancreatic injury in COVID-19 patients.

As previously discussed, ample evidence supporting an association between COVID-19 and abnormal LFTs suggests that SARS-CoV-2 infection might directly or indirectly cause liver injury [126]. The extent of this liver damage correlated with the severity of COVID-19 and the mortality risk [10,67,68,69,70,71,72]. From a pathophysiological perspective, direct cellular damage mediated by SARS-CoV-2 is a primary mechanism of liver disease initiation. However, the presence of the ACE2 receptor in the liver, necessary for this process, remains a subject of contrasting evidence [81,82,83]. Dysregulation of the immune response, marked by increased levels of inflammatory markers in COVID-19 patients, is believed to contribute to hepatic injury [84,85,86,87]. Likewise, hepatocyte death could be mediated by hypoxic and ischemic events resulting from alteration of the coagulation cascade, lipotoxicity, ATP depletion, free radical production, and systemic pro-inflammatory state. In addition, it is essential to consider the potential role of drugs with hepatotoxic effects, such as acetaminophen, or those associated with alteration of LFTs, such as tocilizumab [100,101] or some antivirals [102,103].

Regarding the association between NAFLD/NASH and COVID-19, some authors have studied the impact of the pandemic on this group of patients. In the study conducted by Rivera-Esteban et al. [127], where 354 compensated patients with NAFLD cirrhosis were included, it was observed that after one year of follow-up, the subjects had a 3.9% increase in the rate of the first liver-related event during the pandemic period, as well as a higher rate of metabolic worsening (38.4% vs. 46.1%; *p* = 0.041). On the other hand, Canillas et al. [128] conducted an observational study that includes a retrospective review of factors linked to abnormal LFTs during COVID-19 and a blood test, transient elastography, and liver biopsy in individuals with persistent abnormal LFTs. During COVID-19, abnormal LFTs were associated with infection severity. The authors reported that abnormalities resolved in >80% of patients at follow-up, and no association was detected between abnormal LFTs at admission and follow-up. Most individuals with abnormal LFTs at follow-up had noninvasive and histologically confirmed fatty liver disease.

As previously stated, NAFLD or NASH are a significant risk factor for COVID-19 aggravation [118]. These entities are characterised by inducing a chronic inflammatory state, which might be critical in exacerbating the SARS-CoV-2-induced “cytokine storm” [129]. This chronic inflammation causes macrophage polarisation to the M1 phenotype, decreasing the anti-inflammatory activity of M2 macrophages. This phenomenon generates a molecular activation cascade that ends in the inducement of hepatic fibrosis in patients with NAFLD/NASH [115,116,117], representing an independent risk factor for severity in patients with COVID-19 [117].

Obesity is also a chronic inflammatory condition that negatively impacts immune function; therefore, its presence in NAFLD/NASH patients may worsen the prognosis of COVID-19 [120,130]. Thus, NAFLD/NASH patients may be prone to COVID-19 and its complications by having a defective immune response that increases the risk of SARS-CoV2 infection. This, in turn, could increase the risk of NAFLD progressing to NASH or, at least, cause hepatic alterations through a direct attack on the hepatocytes. Since NAFLD/NASH previously injured the liver, hepatocytes would increase their expression of ACE2 and facilitate the entry of the virus into these cells [108,111,118,131].

However, this has not been confirmed, as ACE2 is undetectable in hepatic stellate cells and myofibroblasts. Conversely, there are other cells where the presence of this enzyme has been confirmed. Such is the case in monocyte-derived macrophages and alveolar macrophages, thus raising the possibility of SARS-CoV-2 achieving its detrimental effects on the liver via Kupffer cells or through viral invasion of circulating monocytes. It is considered that hepatic lesions can lead to Kupffer cell depletion, which is then replaced by circulating monocytes that will go on to form a new population of resident tissue macrophages [121,132].

SARS-CoV-2 causes microvesicular and macrovesicular steatosis through lipid metabolism and mitochondrial activity dysregulation, which may result in disease synergy in COVID-19 and NAFLD/NASH patients [121,122]. It can also indirectly damage the liver through an uncontrolled immune response, hypoxia, or drug-induced hepatotoxic effects, making patients with NAFLD/NASH more susceptible [123,124].

Although this narrative review of the literature provides a synthesised theoretical framework to support future research, its findings should be interpreted with caution due to certain limitations. While the methodology utilised to seek, select, and incorporate the studies was well-defined, it failed to adhere to a rigorous and systematic protocol. Conversely, the data were subjected to qualitative analysis rather than statistical analysis. Consequently, the findings of each study must be interpreted within its specific context and scope, rendering them unsuitable for establishing comparisons across investigations. This limitation stems from the fact that each study employed unique criteria for patient selection, evaluation, and outcomes.

## 6. Conclusions

It has been demonstrated that SARS-CoV-2 infection damage is not only exclusively limited to the respiratory system but also capable of a multisystemic disease. Epidemiologic evidence supports the relationship between COVID-19 and pancreatic injury, primarily due to a direct viral invasion of pancreatic cells, including β cells, and backing a possible association between SARS-CoV-2 and diabetes mellitus. Other secondary mechanisms may involve the immune dysregulation leading to the cytokine storm and certain drugs used for COVID-19 treatment.

Furthermore, plenty of evidence sustains the hypothesis that SARS-CoV-2 infection could be either a direct or an indirect cause of liver injury through a multifactorial complex, including defects in the immune response and the hepatotoxic effects of the drugs frequently used in COVID-19. The association seems bidirectional, as existing hepatic pathologies, such as NAFLD or NASH, are substantial risk factors for COVID-19 worsening.

Even if the current understanding of the pathophysiological mechanisms underlying the development of pancreatitis and liver injury linked to COVID-19 is still scarce, the present evidence argues for the cautious monitoring of pancreatic and liver function parameters in patients with COVID-19.

## Figures and Tables

**Table 1:** Cases of acute pancreatitis in COVID-19 patients: COVID-19 as a possible aetiology.

Authors [Ref]	Study Design	Total Number of Patients	Number of Patients with AP	AP, According to RAC	Observations
Kataria et al. [15]	Case report	1 Female (49Y)	-	1	CT scan: edematous pancreas with diffuse enlargement ^†^
Alves et al. [16]	Case report	1 Female (56Y)	-	1	CT scan: tail parenchymal enlargement and surrounding retroperitoneal fat stranding ^†^
Kumaran et al. [17]	Case report	1 Female (67Y)	-	1	CT scan: necrotising Pancreatitis ^†^
Rabice et al. [18]	Case report	1 Female (36Y) (P)	1	-	Lipase was at 875 U/L; an abdominal ultrasound did not show the pancreas due to bowel gas
Cerda-Contreras et al. [19]	Case report	1 Female (72Y) (S)	-	1	Patient received baricitinib * and dexamethasone CT scan: pancreatic oedema. No information regarding abdominal pain
Akarsu et al. [20]	Prospective study	316	-	40 (12.6%)	7.9% (*n* = 15) of patients with severe symptoms and 32.5% (*n* = 25) of patients in critical status ^Δ^
Juhász et al. [21]	Systematic review	19	6 (32.5%)	5 (26.3%)	Studies included contained data on 11 COVID-19 patients with AP ^Δ^

NA = not available, CT scan = computerised tomography scan, AP = acute pancreatitis, RAC = Revised Atlanta classification, F = female, Y = years, P = pregnant, S = sedated, Δ = Other potential causes of AP were not thoroughly explored, † = After ruling out other etiologies, COVID-19 is considered as a potential cause, * = potential cause of AP.

**Table 2:** Hyperamylasemia and hyperlipasemia during SARS-CoV-2 infection.

Authors [Ref]	Sample	Patients with Altered Pancreatic Enzymes	Amylase (U/L) (Mean ± SD)	Lipase (U/L) (Mean ± SD)	Observations
Wang et al. [13]	*n* = 52	*n* = 9 (17.3%)	115 ± 25	71 ± 34	Normal Amylase range 0–90 U/L and Normal Lipase range 0–70 U/L
Bruno et al. [14]	*n* = 70	*n* = 6 (8.5%)	338.3 ± 246.6	1509 ± 1163	Hyperlipasemia was defined as an elevated lipase level above the ULN (>393 U/L). All patients meet these criteria.
McNabb-Baltar et al. [22]	*n* = 71	*n* = 9 (12.1%)	NP	151.8 ± 148.4	Hyperlipasemia was defined as an elevated lipase level above the ULN (>60 U/L). Two patients (2.8%) developed hyperlipasemia greater than three times the ULN (>180 U/L).
Barlass et al. [23]	*n* = 83	*n* = 14 (16.8%)	NP	NA	High lipase was defined as levels greater than three times the ULN (>156 U/L)
Rasch et al. [24]	*n* = 38	*n* = 12 (31.6%)	NP	422 (186–1127) *	Patients with lipase activity at least three times greater than the ULN (>180 U/L)
Zhang et al. [25]	*n* = 19	*n* = 19 (100%)	64.3 (56.4–94.6) * (mg/L)	NP	-

NA = not available, NP = not performed, SD = standard deviation, ULN = upper limit of normal, * median (range).

**Table 3:** Summary of findings on meta-analyses on the association between COVID-19 severity and liver injury.

Authors [Ref]/ N° Studies Included	Participants	Liver Function Tests (Mean Differences between Severe Cases Versus Non-Severe Cases)
Ahmed et al. [67] N° = 27	Total = 8817 Severe cases = 2900 Non-severe cases = 7184	ALT (WMD: 7.19 U/L; 95% CI: 4.90 to 9.48; *p* < 0.001; I^2^ = 69%) AST (WMD: 9.02 U/L; 95% CI: 6.89 to 11.15; *p* < 0.001; I^2^ = 73%) BIL (WMD: 1.78 μmol/L; 95% CI: 0.86 to 2.70; *p* < 0.001; I^2^ = 82%) ALB (WMD: −4.16 g/L; 95% CI: −5.97 to −2.35; *p* < 0.001; I^2^ = 95%)
Abdulla et al. [68] N° = 12	Total = 6976 Subgroup analysis: Severe cases = 131 Non-severe cases = 334	ALT (WMD: 31.66 U/L; 95% CI: 25.07, 38.25; *p* < 0.0001; I^2^ = 55.64%) AST (WMD: 41.79 U/L; 95% CI: 32.85, 50.72; *p* < 0.0001; I^2^ = 51.43%) BIL (WMD: 9.97 μmol/L; 95% CI: 8.46 to 11.48; *p* < 0.0001; I^2^ = 98%) ALB (WMD: 34.03 g/L; 95% CI: 27.42 to 40.63; *p* < 0.0001; I^2^ = 96.83%)
Parohan et al. [69] N° = 20	Total = 3428 Severe cases = 1455 Mild cases = 1973	AST (WMD: 8.84 U/L; 95% CI: 5.97 to 11.71; *p* < 0.001) ALT (WMD: 7.35 U/L; 95% CI: 4.77 to 9.93; *p* < 0.001) BIL (WMD: 2.30 mmol/L; 95% CI: 1.24 to 3.36; *p* < 0.001) ALB (WMD: −4.24 g/L; 95% CI: −6.20 to −2.28; *p* < 0.001)
Wu and Yang [70] N° = 13	Total = 3722 Severe cases = 721 Non-severe cases: 1968 Dead/Alive: 285/748	AST (WMD:3.35 U/L; 95% CI: 2.07 to 4.64; *p* < 0.001) BIL (WMD: 1.18 μmol/L; 95% CI: 0.78 to 1.58; *p* < 0.001) Liver dysfunction and mortality of COVID-19 patients: OR = 1.98; 95% CI: 1.39 to 2.82; *p* = 0.0002 Liver dysfunction and severity of COVID-19 patients: AST (OR = 4.48; 95% CI 3.24 to 7.21; *p* < 0.001) BIL (OR = 1.91; 95% CI: 1.40 to 2.60; *p* < 0.001)
Shokri Afra H. et al. [71] N° = 24	4246 patients Severe cases: 1635 Non-severe cases: 2611	ALT (SMD: 1.40 U/L; 95% CI: 0.93 to 1.88; *p* < 0.05; I^2^ = 96.5%) AST (SMD: 2.11 U/L; 95% CI: 1.40 to 2.83; *p* < 0.05; I^2^ = 97.9%) BIL (SMD: 1.08 μmol/L; 95% CI: 0.44 to 1.72; *p* = 0.001; I^2^ = 97.7%) ALP (SMD: 0.31; 95% CI: −1.57 to 2.20; *p* = 0.74)
Youssef et al. [72] N° = 20	Total = 3428 patients Severe cases = 1241 Non-severe cases: 2187	Prothrombin time (SMD: 0.69; 95% CI: 0.57 to 0.81; *p* < 0.001) AST (SMD: 0.36; 95% CI: 0.27 to 0.44; *p* < 0.001) ALT (SMD: 0.44; 95% CI: 0.35 to 0.52; *p* < 0.001) BIL (SMD: 0.40; 95% CI: 0.31 to 0.50; *p* < 0.001) ALB (SMD: −0.68; 95% CI: −0.7 to −0.58; *p* < 0.001)
Kumar-M et al. [10] N° = 128	-	BIL (SMD: 0.43 μmol/L; 95% CI: 0.26 to 0.61; *p* < 0.05; I^2^ = 66%) ALB (SMD: −1.05 g/L; 95% CI: −1.27 to −0.83; *p* < 0.05; I^2^ = 77%) GGT (SMD: 0.76; 95% CI: 0.40 to 1.12; *p* < 0.05; I^2^ = 82%) Liver dysfunction and severity of COVID-19 patients: BIL (RR = 1.82; 95% CI: 1.22 to 2.73; *p* < 0.05; I^2^ = 66%) ALB (RR = 2.65; 95% CI: 1.38 to 5.07; *p* < 0.05; I^2^ = 79%) GGT (RR = 2.31; 95% CI: 1.6 to 3.33; *p* < 0.05; I^2^ = 55%)

ALT: alanine aminotransferase; AST: aspartate aminotransferase; BIL: bilirubin levels; ALB: albumin levels; ALP: alkaline phosphatase; GGT: gamma-glutamyltransferase; WMD: weighted mean differences; SMD: standardised mean differences; OR: odds ratio; RR: relative risk CI: confidence interval; I^2^: heterogeneity.
